# The Microbiome Connection: A Common Pathway Linking Cancer and Heart Failure

**DOI:** 10.3390/biomedicines13061297

**Published:** 2025-05-25

**Authors:** Ioannis Paraskevaidis, Elias Tsougos, Christos Kourek

**Affiliations:** 1 Medical School of Athens, National and Kapodistrian University of Athens, 15772 Athens, Greece; iparas@otenet.gr; 2 Department of Cardiology, Hygeia Hospital, 15123 Athens, Greece; tsougos@yahoo.com

**Keywords:** heart failure, cardiovascular disease, cancer, tumors, microbiome

## Abstract

In humans, heart failure (HF) and cancer are among the leading causes of morbidity and mortality. A growing body of evidence highlights a bidirectional relationship between these conditions, underpinned by shared risk factors and overlapping pathophysiological pathways. This review aims to explore the emerging role of the intestinal microbiome as a common mechanistic link between HF and cancer. Specifically, we examine how microbial dysbiosis and its metabolic products—such as trimethylamine N-oxide (TMAO), short-chain fatty acids (SCFAs), bile acids, lipopolysaccharides (LPS), and branched-chain amino acids (BCAAs)—contribute to inflammation, immune dysregulation, oxidative stress, and metabolic dysfunction. These mechanisms promote multiorgan impairment and establish a vicious cycle that fuels both tumorigenesis and cardiac deterioration. HF, cancer, and the gut microbiome are not isolated entities but are deeply interconnected through shared biological mechanisms—including chronic inflammation, microbial dysbiosis, immune and neurohumoral modulation, and metabolic derangement. These findings support the concept of a microbiome-centered axis involving the gut, heart, and tumors, which may underlie many chronic disease processes. Understanding these interactions may provide novel insights into disease pathogenesis and uncover promising therapeutic targets that leverage microbiome modulation to prevent or treat HF, cancer, and other systemic diseases.

## 1. Introduction

Heart failure (HF) and cancer are two of the most pressing global health challenges of the 21st century. HF affects over 64 million people worldwide and remains a leading cause of hospitalization and death, particularly among the aging population. Its global burden continues to rise due to increased longevity and improved survival after acute cardiovascular events. One-year mortality rates following hospitalization for acute HF reach 20–30%, and the condition is associated with high readmission rates and reduced quality of life [1].

Cancer is responsible for nearly 10 million deaths annually, with projections estimating over 28 million new cases worldwide by 2040. This increase is driven by aging populations and lifestyle-related risk factors [2]. In Europe, cancer accounts for approximately one in four deaths, making it the second leading cause of death after cardiovascular diseases. In 2020, cancer caused nearly 1.9 million deaths across the continent, representing about 25% of all deaths [3].

Notably, a growing number of patients are being diagnosed with both HF and cancer—either sequentially or concurrently—suggesting shared pathophysiological mechanisms and a potential bidirectional relationship [4]. Patients with HF may develop cancer [4,5,6], while cancer patients—especially those undergoing antitumor treatments—are at increased risk of developing HF [7,8]. Therefore, long-term follow-up of both groups is recommended [9,10].

These two conditions share common risk factors, including smoking, hypertension, metabolic imbalance, and genetic alterations. They also follow similar pathophysiological mechanisms, such as activation of the neurohumoral system (including the sympathetic and parasympathetic nervous systems and the renin-angiotensin-aldosterone system), heightened inflammation, and increased production of free radicals. Dysregulation of these pathways disrupts homeostasis, impairs structural and functional integrity, and weakens cellular and tissue defense mechanisms.

The term microbiota refers to various microorganisms, including bacteria, viruses, and fungi, that inhabit different parts of the human body, such as the skin, oral and nasal cavities, stomach, and especially the intestines. In contrast, the microbiome encompasses both the microbiota and their interactions with the host [11]. These microorganisms are essential to human health, supporting metabolic, immune, and inflammatory balance, as well as regulating obesity-related disorders [12,13,14].

In patients with HF and/or cancer, the microbiota and its metabolic products are significantly altered due to an impaired intestinal environment. This disruption, known as dysbiosis, is marked by structural and functional changes and increased intestinal permeability [15,16]. These alterations are not incidental; they are increasingly recognized as active contributors to disease development. The microbiome influences host physiology through immune modulation, metabolic regulation, and communication with the nervous and endocrine systems [17,18,19]. Many of these pathways such as chronic inflammation, oxidative stress, and immune dysfunction are common to both HF and cancer. Given its regulatory role and ability to affect distant organs through microbial metabolites, the microbiome is emerging as a compelling common link between the two diseases.

Thus, HF, cancer, and an altered microbiome are interconnected, sharing common pathophysiological mechanisms such as systemic inflammation, neurohumoral activation, oxidative stress, insulin resistance, and metabolic dysregulation. These processes are often driven by harmful microbial metabolites, including trimethylamine N-oxide (TMAO), lipopolysaccharides (LPS), branched-chain amino acids (BCAAs), and secondary bile acids, which promote endothelial dysfunction, immune activation, mitochondrial stress, and tissue remodeling. By influencing these shared mechanisms, a disrupted microbiome plays a pivotal role in the onset and progression of both cardiovascular and oncologic diseases.

The objective of this review is to explore the microbiome as a shared mechanistic link between HF and cancer, emphasizing the role of microbial metabolites and common pathophysiological pathways such as inflammation, dysbiosis, immune modulation, and metabolic dysfunction.

## 2. The Microbiome in Immunological, Metabolic, and Cardiovascular Homeostasis

The gut microbiome is a key regulator of human health, exerting significant influence on immune, metabolic, and cardiovascular homeostasis. It plays a crucial role in educating and modulating the host immune system by promoting the development of gut-associated lymphoid tissue (GALT), maintaining mucosal barrier integrity, and regulating inflammatory responses [20,21,22]. Commensal microorganisms help maintain immune tolerance to non-pathogenic antigens while ensuring robust defense mechanisms against harmful pathogens.

From a metabolic perspective, the microbiome enhances nutrient absorption, synthesizes essential vitamins, and produces important metabolites such as short-chain fatty acids (SCFAs), bile acids, and amino acid derivatives [23]. Among SCFAs, butyrate and propionate are particularly notable for their anti-inflammatory properties, ability to improve insulin sensitivity, and role in supporting energy balance [24]. Through these functions, the gut microbiota helps regulate body weight, glucose metabolism, and lipid levels.

Cardiovascular health is also strongly influenced by the microbiome. Microbial metabolites affect endothelial function, vascular tone, and the risk of atherosclerosis [25,26]. SCFAs contribute to vascular protection and help lower blood pressure [27], whereas dysregulated production of other metabolites, such as TMAO, can promote vascular inflammation and atherogenesis [28]. By maintaining metabolic balance, reducing oxidative stress, and modulating systemic inflammation, a healthy microbiome supports cardiovascular resilience.

Understanding these homeostatic functions is essential for appreciating how microbiome disturbances contribute to the development and progression of complex diseases such as HF and cancer.

Although experimental studies have described the role of the gut microbiome in maintaining immune, metabolic, and cardiovascular balance, longitudinal studies in humans remain limited. Future research should aim to characterize microbial profiles across diverse populations and investigate how specific microbiome configurations influence disease risk or resilience over time.

## 3. The Microbiome Interplay

The microbiome can exert both oncogenic and tumor-suppressive effects. Its oncogenic potential is well documented in several types of cancer. For example, *Helicobacter pylori* is implicated in gastric cancer; hepatitis B and C viruses in liver cancer; human papillomavirus in cervical and vaginal cancers; Epstein-Barr virus in nasopharyngeal carcinoma and lymphoma; and *Escherichia coli* in colorectal cancer, among others [28]. Conversely, some microbiota—such as butyrate-producing bacteria—have demonstrated protective effects by inhibiting the progression of colorectal cancer [29].

Regardless of whether its effects are oncogenic or protective, the microbiome clearly exerts a profound influence on immune cells and inflammatory processes. This is especially relevant given that the gastrointestinal tract contains the majority of the body’s immune cells. Moreover, microbial metabolites can have effects beyond the gut by entering the enterohepatic circulation, thereby influencing distant organ systems [28].

In HF, reduced cardiac output leads to gut ischemia and congestion—observed in both HF with preserved ejection fraction (HFpEF) and HF with reduced ejection fraction (HFrEF) [30]. These changes disrupt the intestinal microbiota and increase intestinal permeability [16,31]. The resulting alterations: (a) activate immune and inflammatory responses, and (b) stimulate the neurohumoral axis, which contributes to left ventricular remodeling and myocardial fibrosis [30,32]. Additionally, these changes are linked to insulin resistance, obesity, metabolic syndrome [33], and impaired mitochondrial energy metabolism [15,19].

Taken together, these findings highlight how intestinal dysbiosis—characterized by disruption of the normal microbiota and its metabolic products—affects core pathophysiological mechanisms common to both cancer and HF. These include neurohumoral overactivation, chronic inflammation, and disturbances in metabolism and energy production [31,34].

**Figure 1:**
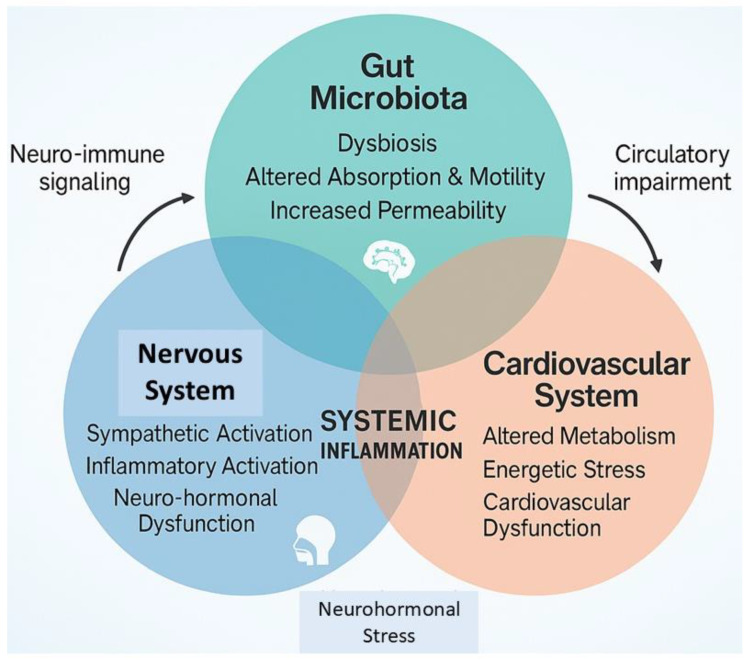
The reciprocal interplay between gut microbiota and the nervous and cardiovascular systems. The altered intestinal homeostatic status initiates neuro-humoral, nervous, and immune system dysfunction, pillars of both cancer and cardiovascular disease pathophysiology.

There is a clear bidirectional relationship between the microbiome, HF, and cancer. Each condition can alter the composition and function of the gut microbiota, which in turn contributes to the development and progression of these diseases, establishing a self-perpetuating cycle (Figure 1).

While compelling evidence links microbiota-derived metabolites to both oncogenesis and cardiovascular dysfunction, much of the current data stems from preclinical studies. Translational research and human interventional trials are urgently needed to validate these causal relationships and to explore strategies for therapeutic modulation of the microbiome.

Table 1 summarizes key microbiome-derived carcinogenic metabolites, their bacterial sources, the pathways they activate, their effects on antitumor immunity, the associated cancer types, and their relationships with cardiovascular dysfunction and heart failure.

### 3.1. Shared Microbial Mechanisms in HF and Cancer

Dysbiosis refers to an imbalance or disruption in the normal composition, diversity, or function of the microbiome. It may involve a reduction in beneficial commensal microbes, an overgrowth of pathogenic species, or a loss of overall microbial diversity. Dysbiosis can be triggered by a variety of factors, including dietary changes, infections, antibiotic use, chronic diseases, and environmental exposures [35,36]. It plays a central role in the pathogenesis of both HF and cancer through shared biological pathways. Dysbiosis is marked by a reduction in beneficial commensals, an overgrowth of pathogenic species, and altered microbial metabolism, which together promote systemic inflammation, oxidative stress, and immune dysfunction [15,35,36,37].

Key microbial metabolites such as TMAO, LPS, SCFAs, secondary bile acids, BCAAs, and phenylacetylglutamine (PAGln) exert profound systemic effects. For example, TMAO, derived from dietary choline and carnitine by gut bacteria and converted in the liver via flavin-containing monooxygenases, promotes vascular inflammation, endothelial dysfunction, platelet hyperreactivity, and adverse cardiac remodeling [38,39,40,41,42,43,44,45,46]. It activates inflammatory pathways including NF-κB and the NLRP3 inflammasome, and has been associated with an increased risk of atherosclerosis, HF, and cancer progression [39,40,43,45].

Similarly, LPS, a product of Gram-negative bacteria such as Enterobacteriaceae, crosses a compromised intestinal barrier and enters systemic circulation. It activates Toll-like receptor 4 (TLR4), induces macrophage polarization, and drives the release of proinflammatory cytokines—key processes that contribute to myocardial inflammation, vascular dysfunction, and tumor-promoting immune modulation [37,47,48,49,50].

While SCFAs such as butyrate and propionate normally exert anti-inflammatory, antitumor, and insulin-sensitizing effects, their production is often diminished in dysbiotic states [23,24,51,52]. This reduction in protective metabolites, coupled with an increase in harmful ones, results in immune dysregulation, metabolic disturbance, mitochondrial stress, and chronic low-grade inflammation—all of which are common to both cancer and cardiovascular pathology [12,14,15,19,53,54,55].

The pathophysiological impact of these metabolites is not unidirectional. In both HF and cancer, impaired intestinal perfusion, systemic inflammation, and the effects of chemotherapeutic or cardiovascular therapies further exacerbate dysbiosis, creating a vicious cycle of mutual reinforcement [16,30,31,32]. Thus, the gut microbiota is not merely a bystander but an active participant in disease propagation, forming the foundation of a bidirectional, metabolite-driven feedback loop.

This unified perspective underscores the microbiome as a mechanistic bridge between oncologic and cardiovascular disease, and highlights shared therapeutic opportunities. Targeting microbiome-derived metabolites through diet, probiotics, or pharmacological agents may represent a promising strategy to attenuate systemic inflammation, restore metabolic balance, and interrupt disease progression.

While mechanistic pathways involving TMAO, LPS, and SCFAs have been well characterized in preclinical models, their direct causal roles in human disease progression, particularly in the context of concurrent HF and cancer, remain an area of active investigation.

### 3.2. Metabolic/Energetic Status

The intestinal microbiome plays a critical role in regulating host energy balance, metabolic homeostasis, and nutrient utilization. Under normal conditions, commensal microbes enhance carbohydrate fermentation, producing beneficial SCFAs such as butyrate and propionate. These SCFAs serve as energy substrates, maintain epithelial integrity, and exhibit anti-inflammatory and insulin-sensitizing properties [12,14,23,24,51,52]. The microbiome also participates in bile acid metabolism, modulates glucose and lipid profiles, and influences the bioavailability of micronutrients and neurotransmitters [15,23,56,57,58].

In dysbiotic states—commonly observed in both HF and cancer—the microbial production of protective SCFAs diminishes, while harmful byproducts such as TMAO, LPS, and BCAAs increase [15,53,59,60,61]. These changes promote insulin resistance, metabolic inflammation, mitochondrial dysfunction, and impaired oxidative metabolism [15,19,62,63,64].

Dietary composition significantly shapes the metabolic output of the microbiota. High-fat and high-cholesterol diets favor the generation of LPS and TMAO—metabolites strongly associated with atherosclerosis, cardiac fibrosis, and tumor progression [38,39,40,41,42]. Conversely, fiber-rich diets support SCFA production and metabolic resilience [15,51,52]. The microbiome’s metabolic output is also influenced by local factors such as intestinal pH, oxygen levels, and motility, as well as systemic factors including bile and pancreatic secretions, hormonal status, and host genetics [15,65,66,67,68,69].

Thus, the microbiome functions as a metabolic integrator between the gut and peripheral organs. In HF and cancer, its dysregulation contributes to energetic depletion, oxidative stress, and chronic inflammation—hallmarks that accelerate disease progression [15,53,54,55]. Interventions aimed at modulating microbial metabolism—such as dietary modification, probiotics, or targeted pharmacologic agents—may offer novel strategies to restore systemic metabolic balance.

### 3.3. Inflammation/Free Radical Production

The gut microbiota closely interacts with the host immune system and plays a critical role in regulating inflammatory responses at both local and systemic levels. Under normal physiological conditions, the microbiome supports the maturation of lymphoid tissues, maintains epithelial barrier integrity, and promotes immune tolerance by modulating T-cell responses and dendritic cell function [70,71,72,73]. Commensal bacteria also stimulate the expansion of cytotoxic CD8+ T cells, which possess anti-tumor activity, while preserving mucosal immune homeostasis [74,75].

However, during dysbiosis, pathogenic microbes and their toxic metabolites—such as lipopolysaccharides (LPS) and reactive nitrogen species—can dominate the gut environment. This shift impairs immune defense mechanisms and initiates chronic inflammation. The resulting disruption of mucosal immunity increases cytokine production and promotes abnormal epithelial cell proliferation, contributing to carcinogenesis [76,77,78]. Dysbiosis has also been linked to the development of cancers beyond the gut through systemic inflammatory pathways and microbial translocation [79,80,81].

Importantly, microbes originating in the gut have been identified within tumor tissues, forming what is known as the intratumoral microbiota. These microbes, often residing within immune cells, interfere with autophagy and immune surveillance, thereby promoting tumor progression [82,83,84]. At the same time, inflammation and microbial imbalance trigger systemic endocrine and neurohormonal changes—including insulin resistance, activation of the renin-angiotensin-aldosterone system (RAAS), and increased oxidative stress—all of which are key contributors to the development of both HF and cancer [33,85,86,87,88].

Free radicals, including reactive oxygen species (ROS) and reactive nitrogen species (RNS), are major mediators of inflammation and cellular injury. While they serve important roles in signaling and immune defense at low concentrations, excessive production—common in dysbiotic states—results in lipid peroxidation, protein oxidation, and DNA damage [89,90,91]. In the heart, oxidative stress promotes myocardial cell apoptosis, necrosis, fibrosis, and mitochondrial dysfunction. These changes impair cardiac contractility and drive the progression of HF [91,92,93,94]. In cancer, free radicals contribute to genomic instability, damage tumor suppressor genes, and promote a tumor-friendly microenvironment, particularly under hypoxic conditions [95,96,97,98].

These interconnected mechanisms—chronic inflammation, immune dysregulation, and oxidative stress—represent a shared pathogenic link between dysbiosis and both cardiovascular and oncologic diseases. Although these associations are supported by both experimental and clinical data, the exact sequence of events and causal relationships remain uncertain. Further longitudinal studies are needed to determine whether targeting the microbiome can reduce oxidative and inflammatory damage in HF and cancer.

Although dysbiosis-induced immune dysregulation and oxidative stress are strongly implicated in preclinical models of both cancer and cardiovascular disease [89,90,91,92,93], the temporal sequence and clinical causality of these processes in humans are not yet fully delineated.

### 3.4. The Multiciliary Axis

**Figure 2:**
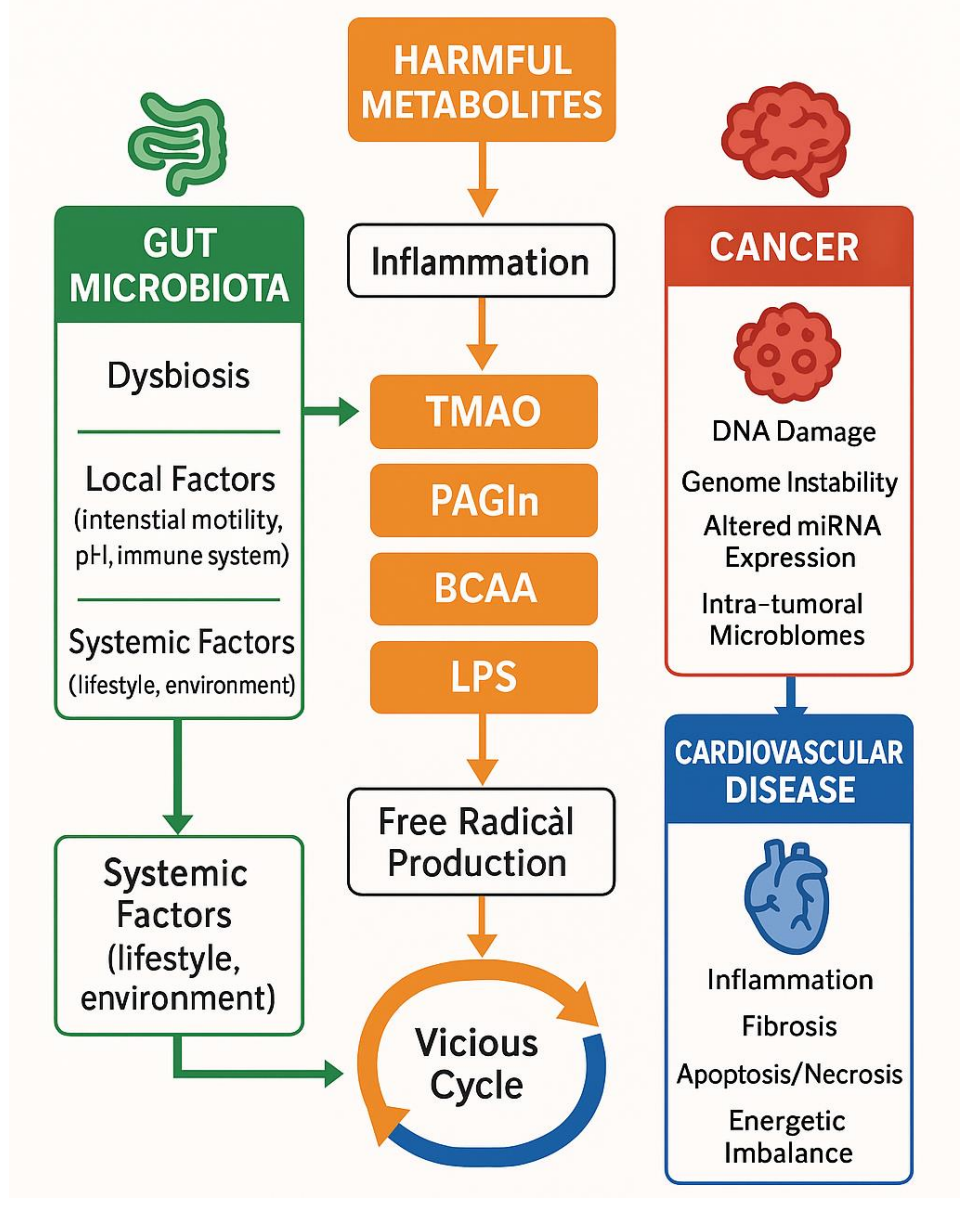
Pathophysiological concepts for microbiome—cancer—cardiovascular diseases interplay. BCAA: branched-chain amino acids, CV: cardiovascular diseases, LPS: lipopolysaccharides, PAGLn: phenylacetylglutamine, TMAO: Trimethylamine N-Oxide.

The microbiome plays a regulatory role in neurogenesis, myelination, glial cell function, synaptic pruning, and blood–brain barrier permeability [18]. Communication with the central nervous system (CNS) is bidirectional, involving metabolic, endocrine, neurological, and immune pathways that influence both the onset and progression of various diseases [18,99]. This communication—often referred to as the “gut–brain axis”—occurs via both neural and humoral routes. Microbiome-derived signals reach the brain through stimulation of the enteric nervous system and the vagus nerve [100], or through the systemic circulation, which transports microbial metabolites—both beneficial and harmful—across the blood–brain barrier [101,102] (Figure 2).

Increased intestinal permeability allows microbial products such as LPS and SCFAs to activate both peripheral and central immune cells, promote cytokine release, and induce neuroinflammation. These processes affect CNS function and contribute to disease pathophysiology. Microbial products, including SCFAs, bile acid derivatives, neurotransmitter agonists, tryptophan metabolites, serotonin, and catecholamines, can modulate host metabolic and inflammatory responses, contributing to the development and progression of both cancer and cardiovascular diseases [103,104,105,106]. The inflammatory response triggered by harmful microbial stimuli activates immune cells and promotes cytokine release, which directly impacts CNS function [99]. This axis highlights an indirect mechanism by which the microbiome influences the nervous system and broader human physiology.

According to the International Cancer Microbiome Consortium, there is currently no direct evidence that the human commensal microbiome is a key determinant in the etiology of cancer [107]. While some cancers such as those associated with bacterial vaginosis and co-infection with HIV or human papillomavirus may involve a more direct microbial role, it appears that toxic products from a dysregulated microbiome are the primary contributors to cancer development. These metabolites disrupt host homeostasis, promote systemic inflammation and neurohumoral activation, and drive pathological processes that can facilitate cancer progression even at distant sites.

**Figure 3:**
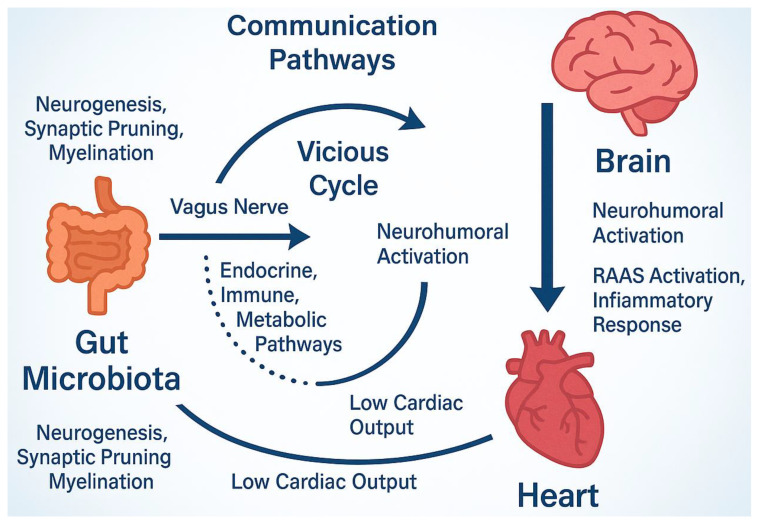
Microbiome nervous system communication through the gut–brain axis. This axis using metabolic, endocrine and other pathways interfere with neurogenesis, myelination etc. The altered microbiota environments activate RAAS system that, in turn, promote free radical production and inflammatory response, the basic concept of HF syndrome. Accordingly, low cardiac output further aggravates these actions and stimulate central nervous system constituting thus a vicious cycle with harmful effects. RAAS; Renin-angiotensin-aldosterone system.

In HF, the nervous system is also significantly affected. Chronic cerebral hypoperfusion, inflammation, oxidative stress, and overactivation of the RAAS are key factors. RAAS is active in several organs, including the brain, heart, lungs, and intestines, and functions in an integrated manner to regulate homeostatic processes such as glycemic control and electrolyte balance [108]. When the gut microbiota and its metabolic outputs are pathologically altered, these regulatory mechanisms are disrupted. This disruption promotes cardiac fibrosis, cellular apoptosis and necrosis, and ultimately, the progression of HF [109,110] (Figure 3).

In addition, conditions such as insulin resistance, obesity, and metabolic syndrome [33], which are central to cardiovascular disease pathophysiology [19,31], are frequently present. In HF, reduced cardiac output and venous congestion impair intestinal function and promote dysbiosis. This microbial imbalance leads to excessive LPS production, which further increases intestinal permeability. The resulting endotoxemia drives systemic inflammation and activates immune and neurohumoral pathways [48,49,50], thereby exacerbating the severity of HF.

Clearly, a vicious cycle exists between the microbiome, HF, and the nervous system. These systems share overlapping pathophysiological mechanisms [100], and their dysfunction contributes to complications such as cognitive impairment [111]. Interestingly, the microbiome can also interact with the host’s reward pathways (e.g., the mesolimbic system) and modulate the effects of noradrenaline on the bone marrow, thereby enhancing antitumor immunity. Furthermore, the use of probiotics as adjunctive cancer therapy has shown potential to modulate the microbiome, improve psychological well-being, and slow cancer progression [112,113].

There is little doubt that a dynamic, reciprocal interplay exists among the brain, gut, and heart, forming a regulatory axis that governs nutrient absorption, gut motility, intestinal permeability, and broader biochemical, metabolic, and neurohormonal balance. Disruption of this axis compromises these homeostatic processes and may trigger disorders affecting the brain, gut, heart, and even cancer development [100].

Remarkably, this axis appears to be part of a broader, multicentric regulatory network involving multiple organ systems, including the brain (e.g., anxiety, depression), endocrine system (metabolic and hormonal disorders), cardiovascular system (heart disease, thrombosis), lungs (chronic obstructive pulmonary disease), liver (cirrhosis), pancreas (diabetes), and bones (osteoporosis), among others. Dysregulation within this network contributes to the pathogenesis of a wide range of systemic diseases [15].

Although the role of the gut–brain axis in disease is increasingly recognized, the precise pathways through which microbiome-induced neuroinflammation contributes to cancer and HF remain incompletely understood. Advanced imaging techniques and biomarker-based studies are needed to clarify the temporal and mechanistic aspects of this complex interplay.

Much of the current understanding of the gut–brain–heart axis stems from animal studies and observational human data. The precise pathways through which microbiome-driven neuroinflammation influences cancer and HF progression in humans remain largely hypothetical and warrant further clinical validation.

Is there a connection among these clinical states and genomic alterations?

## 4. Microbiome Relation to Genomic Mutation and Instability

The connection between cancer and an altered microbiome or metabolic environment, specifically dysbiosis, is well recognized [98,114]. Three key mechanisms explain the interplay between the microbiome, its metabolites, and the initiation and progression of cancer: inflammation, impaired intestinal permeability, and genomic damage. The first two mechanisms have been discussed previously. The third involves direct interactions between the microbiome and host DNA, as dysbiotic microbial communities and their toxic metabolites promote DNA damage, a phenomenon observed in both cancer and cardiovascular diseases.

DNA is a dynamic molecule that constantly undergoes replication and recombination. The fidelity of these processes depends on the cell’s ability to detect and repair abnormalities. However, when the frequency or intensity of DNA-damaging factors exceeds the repair capacity, lesions such as base mismatches, single- or double-strand breaks, and DNA adducts can occur. These abnormalities lead to defective sequences and the production of dysfunctional proteins. The accumulation of such DNA damage is a central driver of cellular mutations and, ultimately, tumorigenesis [114,115].

Microbes residing in various organs, especially the gastrointestinal tract, have also been detected within tumors, forming what is known as intratumoral microbiota. These microbial populations are closely associated with cancer development [116]. Intratumoral microbiomes, particularly those located near human leukocyte antigens (HLA-I and HLA-II), differ significantly from the microbiota of adjacent healthy tissue and vary across different tumor types [117,118,119].

Microbiota-induced DNA damage can occur either directly, as described, or indirectly through increased production of free radicals [120]. Reactive oxygen species (ROS) and reactive nitrogen species (RNS) can modify DNA bases; for instance, through the formation of 8-hydroxy-deoxyguanosine, which results in G→T transversions. These mutations contribute to malignancy by disrupting tumor suppressor genes such as *p53*, stabilizing hypoxia-inducible factors (HIFs), and activating transcription factors like nuclear factor kappa B (NF-κB) and activator protein-1 (AP-1) [121].

Beyond genetic mutations, microbiome dysbiosis can also promote disease through epigenetic modifications. These include chemical alterations to DNA and histones, such as methylation and acetylation, that influence gene expression without altering the DNA sequence itself [122,123,124]. Certain microbial metabolites, particularly SCFAs (e.g., butyrate) and secondary bile acids, can modify the activity of DNA methyltransferases (DNMTs) and histone deacetylases (HDACs), thereby altering DNA methylation patterns [125]. Chronic inflammation and oxidative stress triggered by dysbiosis may promote hypermethylation of tumor suppressor gene promoters or global DNA hypomethylation—both hallmarks of cancer development [126,127]. Changes in histone acetylation can further affect chromatin accessibility and gene transcription, influencing key processes such as cell cycle regulation, apoptosis, and inflammation [127].

Although the role of microbiome-induced epigenetic alterations in HF is less well understood, emerging evidence suggests that oxidative and inflammatory environments driven by dysbiosis can induce epigenetic reprogramming in cardiac tissue. This may contribute to myocardial remodeling, fibrosis, and progression of HF [122,124]. Thus, epigenetic changes represent a crucial mechanistic link between microbiome dysbiosis, oncogenesis, and cardiac dysfunction.

In the context of cardiovascular disease, although the underlying mechanisms remain incompletely defined, gene mutations resulting in abnormal protein expression are increasingly recognized as contributing factors [128,129]. Each individual carries unique genomic variants, including single nucleotide polymorphisms and larger chromosomal abnormalities, which account for approximately 85% of the known genetic variation associated with disease susceptibility [130,131].

These variants can shape disease phenotypes and, conversely, the phenotype may influence microbiome composition. Altered gene expression can directly or indirectly impact microbial populations, which may, in turn, influence disease manifestation. This highlights a strong association between genetic variation and the development of diseases such as cancer and cardiovascular conditions [132].

Notably, associations have been documented between gut microbiota or their metabolites and HF, as well as with its major risk factors, including diabetes, hypertension, myocarditis, myocardial ischemia, arrhythmias, and both hypertrophic and dilated cardiomyopathies [133]. MicroRNAs (miRNAs), small, non-coding RNAs that regulate gene expression by modulating mRNA degradation and translational repression, play vital roles in processes such as cell differentiation, proliferation, and apoptosis [134]. A bidirectional relationship exists between miRNAs and the microbiome: miRNAs can influence microbiome composition and activity, while the microbiome can affect host miRNA expression through its metabolic and inflammatory effects [120,135].

This reciprocal interaction significantly shapes the development of various diseases, including cancer and cardiovascular disorders.

While the epigenetic influence of the microbiome on cancer development is becoming clearer, its role in HF remains underexplored. Future research should focus on elucidating how microbiome-driven DNA methylation and histone modifications contribute to cardiovascular disease. Epigenome-wide association studies conducted in well-characterized patient cohorts could offer valuable insights.

## 5. Insulin Resistance, Hyperinsulinemia, and Their Link to the Microbiome

Insulin resistance, characterized by impaired glucose uptake and utilization, is a central feature of metabolic syndrome, which is strongly linked to both cancer and cardiovascular disease [136]. Hyperinsulinemia—resulting from compensatory pancreatic insulin secretion—fosters a pro-inflammatory, pro-oxidant, and pro-growth environment, conducive to tumor progression and myocardial remodeling. Emerging evidence highlights the gut microbiome as a key modulator of insulin sensitivity, through direct and indirect mechanisms involving microbial metabolites, immune activation, and intestinal permeability [137].

The gut microbiota influences systemic metabolism via SCFAs, LPS, TMAO, and BCAAs. For example, SCFAs such as butyrate have anti-inflammatory and insulin-sensitizing properties [138], while BCAAs and LPS are associated with metabolic inflammation and IR [139,140]. In HF, congestion and gut ischemia promote dysbiosis, favoring Gram-negative bacterial overgrowth and LPS translocation, which activates TLR4 signaling and contributes to systemic insulin resistance [141,142].

In cancer, hyperinsulinemia supports tumor growth by activating insulin and IGF-1 receptors, promoting mitogenic signaling via PI3K/Akt and MAPK pathways [143,144,145]. In parallel, IR impairs mitochondrial efficiency and oxidative metabolism, both of which are disrupted in cancerous and failing myocardial cells. This metabolic derangement is exacerbated by dysbiosis-induced systemic inflammation and oxidative stress, creating a vicious cycle where microbiota, metabolism, and immune responses reinforce disease progression.

Hyperinsulinemia may modulate the tumor microenvironment, impair immune surveillance, and alter drug metabolism—effects that are influenced by microbiota-derived metabolites [146]. For instance, TMAO, produced from dietary choline by gut microbes, is elevated in IR states and is associated with both atherosclerosis and tumor angiogenesis [147].

Intervening on the gut microbiome may attenuate insulin resistance and, by extension, mitigate progression of HF and cancer. Prebiotics, probiotics, and dietary interventions (e.g., Mediterranean diet, fiber-rich intake) have been shown to restore microbial balance and improve insulin sensitivity [148]. Furthermore, emerging strategies such as fecal microbiota transplantation (FMT) and targeted microbial metabolite modulation (e.g., SCFA enhancement, LPS inhibition) may hold promise [149].

Metformin—a cornerstone antidiabetic drug—also exerts microbiome-modulating effects, increasing the abundance of *Akkermansia muciniphila*, a bacterium associated with improved metabolic outcomes [150]. Notably, metformin has shown antitumor effects in epidemiological studies, further linking insulin pathways, microbiota, and cancer biology.

Although associations between dysbiosis and insulin resistance are well-documented, it remains uncertain whether microbiome modulation can consistently reverse insulin resistance in clinical settings. Interventional studies employing probiotics, dietary fiber, or fecal microbiota transplantation are needed to test this therapeutic potential.

## 6. Microbiome Stabilizing Strategies

Several pharmacological and non-pharmacological interventions are essential in the management of both cancer and cardiovascular diseases. Non-pharmacological strategies, such as exercise training, lifestyle modifications, and adherence to a Mediterranean diet, have demonstrated beneficial effects on these conditions [151]. While their positive impact is well documented, potential adverse effects have also been reported [152,153]. These interventions can influence the composition and bioavailability of the intestinal microbiome, thereby affecting metabolic processes, immune cell function, and other host responses.

For example, excessive consumption of high-fat foods can increase the levels of harmful microbial metabolites such as TMAO, LPS, PAGIn, and phenylacetylglycine, all of which are associated with adverse health outcomes [154,155,156,157,158].

Beyond lifestyle interventions, both cardiovascular disease and cancer require pharmacological treatment. However, it is important to recognize that an altered gut microbiome can interfere with drug pharmacokinetics and pharmacodynamics [159,160,161,162]. For instance, the effectiveness of β-blockers [162,163], sodium-glucose cotransporter-2 (SGLT2) inhibitors [164,165], and RAAS inhibitors [110,166] may be reduced in the presence of dysbiosis, potentially leading to suboptimal therapeutic outcomes.

A similar phenomenon is observed with many anticancer therapies. The microbiome and its metabolites—through metabolic, immune (both innate and adaptive), epigenetic, and inflammatory pathways—can influence the effectiveness of immunotherapy [167]. As noted, “the gut microbiota may interact with oncogenic pathways, including epidermal growth factor receptor (EGFR), vascular endothelial growth factor (VEGF), and Kirsten rat sarcoma viral oncogene homolog (KRAS)” [168]. This highlights how gut microbiome alterations can affect not only cancer development and progression but also the response to cancer treatment.

Changes in the microbiome have also been linked to drug resistance. This may occur through mechanisms involving DNA damage, altered drug metabolism, and modifications of the tumor microenvironment [169]. As a result, toxic microbial byproducts may reduce the efficacy of targeted therapies and contribute to tumor growth and progression [170,171]. Specific examples include:

- Irinotecan, a pro-drug used to treat colorectal cancer, is metabolized into the active compound SN-38, a topoisomerase inhibitor. SN-38 can cause DNA damage and severe, potentially life-threatening toxicity [172].
- Gemcitabine, a nucleoside analog used in multiple cancers, can be inactivated by microbial enzymes that convert it into 2′,2′-difluorodeoxyuridine, thereby reducing its therapeutic effectiveness [173].
- Cyclophosphamide, a widely used chemotherapeutic agent, exerts some of its effects through immune modulation—a process also influenced by the gut microbiome [174].

The interaction between the microbiome and pharmacological agents is now well established, prompting growing interest in stabilizing the microbiome to optimize drug responses. Notably, different tumor types have been associated with distinct microbial profiles [175,176], emphasizing the need for personalized treatment strategies based on an individual’s microbiome composition.

In this context, antibiotic therapy targeting specific bacterial species has shown promise. For example, antibiotics targeting *Bacteroides* species have been linked to improved survival in patients with metastatic renal cell carcinoma receiving first-line VEGF tyrosine kinase inhibitors [177]. However, contradictory results in other cancer types suggest that the broad use of antibiotics may not always be beneficial [178].

An emerging area of interest is the use of probiotics and prebiotics to stabilize the gut microbiota. Probiotics—live microorganisms that support health—can help maintain mucosal integrity, regulate intestinal motility, and suppress pathogenic bacteria [11,179]. For instance:

- Lactobacilli produce antioxidant and anti-angiogenic compounds, reduce DNA damage, and mitigate inflammation [180].
- Bifidobacterium species have demonstrated the ability to activate the innate immune system and exhibit anti-cancer properties, particularly in lung, cervical, and breast cancers [180].
- Inulin and galacto-oligosaccharides (GOS) can stimulate immune responses and have shown anticancer potential [181].

Prebiotics, which promote the growth of beneficial gut bacteria, also exert protective effects by modulating intestinal metabolism [179]. A notable example is GOS, a class of carbohydrates found naturally in breast milk. These compounds enhance the production of beneficial cytokines such as interleukin-8 (IL-8), interleukin-10 (IL-10), and C-reactive protein, while reducing harmful ones such as interleukin-1β (IL-1β) [179].

Recent advances in microbiome research highlight the potential benefits of personalizing interventions based on individual microbial profiles to improve the treatment of both cancer and HF. In the context of HF, targeting the gut microbiome may offer an adjunctive strategy to modulate systemic inflammation, improve metabolic parameters, and restore intestinal barrier function. Dietary interventions such as increased fiber intake and adherence to a Mediterranean diet have been associated with enhanced SCFA production, improved endothelial function, and reduced cardiovascular risk [148,151]. Probiotic supplementation, particularly with Lactobacillus and Bifidobacterium species, has shown promise in small clinical studies by attenuating inflammatory cytokine release and improving left ventricular function [180,182]. Fecal microbiota transplantation, though still experimental in cardiovascular settings, offers a way to re-establish a eubiotic microbial ecosystem and has been shown to reduce insulin resistance and systemic inflammation in metabolic disease models. Integration of these interventions into HF management may be especially relevant for patients with comorbid metabolic syndrome, obesity, or gut-derived inflammation, further helping to attenuate systemic inflammation, improve metabolic profiles, and slow cardiac remodeling [182]. In oncology, specific microbial signatures have been associated with responses to immune checkpoint inhibitors and chemotherapy, suggesting that modulating the microbiome could enhance antitumor immunity and reduce drug resistance [183]. Precision microbiome-based interventions offer the possibility of reducing treatment-related toxicity, enhancing therapeutic efficacy, and preventing disease progression by restoring a balanced host–microbiome interaction tailored to each patient’s unique microbial ecosystem.

Although microbiome-targeted therapies are promising, the optimal strategies for specific patient populations remain undefined. Personalized microbiome interventions, tailored to an individual’s microbial profile and disease phenotype, should be a major focus of future clinical trials.

## 7. Challenges to Be Addressed and Strengths of the Manuscript

Although numerous studies support a link between the intestinal microbiome environment and both cancer and cardiovascular diseases, several critical challenges remain. There is a pressing need to generate robust, high-quality evidence to better understand this relationship. Specifically, longitudinal studies are required to determine whether alterations in the microbiome are a cause or a consequence of cancer and/or cardiovascular diseases.

To draw such conclusions, advancements in diagnostic techniques for accurately characterizing microbiome composition are essential. Current methods, such as analyzing blood or fecal samples to identify microbial biomarkers, are widely used but carry notable limitations. In order to improve accuracy and interpretation, it is imperative to first establish a clear definition of what constitutes normal microbiota [184], and to further stratify microbiome profiles by sex and age [185]. Additionally, expanding and refining existing microbiome databases is necessary to overcome current limitations in reference datasets [186].

Although the gut microbiome has been extensively studied in relation to individual disease states such as HF or cancer, its role as a shared pathophysiological bridge between these two major conditions remains underexplored. This review provides a novel and integrative perspective by examining how microbiome-derived metabolites such as TMAO, LPS, and SCFAs, influence common biological pathways including inflammation, immune modulation, metabolic dysfunction, and oxidative stress. We introduce the concept of a “gut–heart–tumor” axis and propose that microbial dysbiosis represents a unifying mechanism driving both cardiac and oncologic disease progression. Furthermore, this review synthesizes emerging evidence on the bidirectional interplay among the gut, heart, and brain, and explores therapeutic opportunities through microbiome modulation. By bridging cardiology, oncology, and microbiome science, our review offers a comprehensive framework that advances current understanding and suggests novel avenues for prevention and treatment in comorbid disease states.

## 8. Conclusions

In conclusion, HF, cancer, and the gut microbiome are not isolated entities but are deeply interconnected through shared biological mechanisms, including chronic inflammation, microbial dysbiosis, immune and neurohumoral modulation, and metabolic derangement. These findings support the concept of a microbiome-centered axis involving the gut, heart, and tumors, which may underlie many chronic disease processes. Recognizing the microbiome as a dynamic contributor to both cardiac and oncological health opens new frontiers for targeted interventions. Although preclinical studies have established compelling mechanistic links between microbial metabolites and both cardiovascular and oncologic pathology, translation into clinical practice remains in its early stages. Most human data are associative or observational, and causality has not been firmly established. Rigorous longitudinal studies, interventional trials, and multi-omics approaches are essential in order to validate microbiome-derived biomarkers and personalize prevention and treatment strategies in patients with concurrent HF and cancer. Modulating the gut microbiota through diet, probiotics, or pharmacological agents may offer a promising therapeutic avenue to simultaneously address the burden of cancer and cardiovascular disease.

## Figures and Tables

**Table 1:** Microbiome-derived carcinogenic metabolites, responsible bacteria, involved pathways, effects on the immune system, associated cancers, and links with heart failure.

Metabolite	Key Bacteria	Pathways Involved	Effect on Antitumor Immunity	Metabolite	Key Bacteria
Acetaldehyde	*Escherichia coli*, *Klebsiella* spp.	DNA damage, ROS generation	DNA adduct formation, immune suppression	Colorectal, esophageal	Promotes systemic oxidative stress and inflammation
Heterocyclic amines (HCAs)	*Clostridium* spp., *Bacteroides* spp.	Activation of NF-κB, CYP450 enzymes	Induces immune evasion via chronic inflammation	Colorectal, pancreatic	Chronic inflammation leads to vascular dysfunction
Secondary bile acids (e.g., deoxycholic acid)	*Clostridium* spp.	FXR signaling, ROS production	Disruption of immune surveillance, promotes tumor growth	Liver, colorectal	Endothelial dysfunction, promotes cardiac fibrosis
Lipopolysaccharides (LPS)	*Enterobacteriaceae*, *Bacteroides* spp.	TLR4 activation, NF-κB pathway	T-cell exhaustion, macrophage polarization	Multiple (systemic effect)	Drives myocardial inflammation and remodeling
Phenylacetylglutamine (PAGln)	*Proteobacteria* group	Adrenergic receptor signaling	Enhances pro-tumorigenic adrenergic responses	Breast, prostate	Induces platelet hyperreactivity, promotes HF
Trimethylamine N-oxide (TMAO)	*Lachnospiraceae*, *Enterobacteriaceae*	Inflammatory, metabolic pathways	Modulates immune cell metabolism	Colorectal, gastric	Strongly associated with atherosclerosis, HF risk

## Abbreviations

AP-1	Activator Protein-1
BCAA	Branched-Chain Amino Acids
CNS	Central Nervous System
EGFR	Epidermal Growth Factor Receptor
GOS	Galacto-Oligosaccharides
HF	Heart Failure
HIF	Hypoxia-Inducible Factors
HIV	Human Immunodeficiency Virus
HLA	Human Leukocyte Antigens
IL	Interleukin
KRAS	Kirsten Rat Sarcoma
LPS	Lipopolysaccharides
miRNAs	MicroRNAs
NF-κB	Nuclear Factor Kappa B
PAGIn	Phenylacetylglutamine
RAAS	Renin-Angiotensin-Aldosterone System
RNS	Reactive Nitrogen Species
ROS	Reactive Oxygen Species
SCFAs	Short-Chain Fatty Acids
SGLT2	Sodium-Glucose Cotransporter-2
TMAO	Trimethylamine N-oxide
VEGF	Vascular Endothelial Growth Factor
